# Creating research-ready partnerships: the initial development of seven implementation laboratories to advance cancer control

**DOI:** 10.1186/s12913-023-09128-w

**Published:** 2023-02-21

**Authors:** Gina R. Kruse, Erica Hale, Justin E. Bekelman, Jennifer E. DeVoe, Rachel Gold, Peggy A. Hannon, Thomas K. Houston, Aimee S. James, Ashley Johnson, Lisa M. Klesges, Andrea L. Nederveld

**Affiliations:** 1 Division of General Internal Medicine, Massachusetts General Hospital and Harvard Medical School, Boston, MA USA; 2 Department of Internal Medicine, Wake Forest School of Medicine, Winston-Salem, NC USA; 3 Atrium Health Wake Forest Baptist, Winston Salem, NC USA; 4 Penn Center for Cancer Care Innovation at the Abramson Cancer Center, University of Pennsylvania, Philadelphia, PA USA; 5 Department of Family Medicine, Oregon Health & Science University, Portland, OR USA; 6 Kaiser Permanente NW Center for Health Research, Portland, OR USA; 7 OCHIN, Inc., Portland, OR USA; 8 Department of Health Systems and Population Health, University of Washington, Seattle, WA USA; 9 Department of Implementation Science, Wake Forest School of Medicine, Winston-Salem, NC USA; 10 Washington University in St Louis, School of Medicine, Division of Public Health Sciences, St. Louis, MO USA; 11 Department of Family Medicine, University of Washington, Seattle, WA USA; 12 Department of Family Medicine, University of Colorado School of Medicine, Aurora, CO USA

**Keywords:** Implementation science, Partnership-building, Community-engaged research, Cancer control

## Abstract

**Background:**

In 2019–2020, with National Cancer Institute funding, seven implementation laboratory (I-Lab) partnerships between scientists and stakeholders in ‘real-world’ settings working to implement evidence-based interventions were developed within the Implementation Science Centers in Cancer Control (ISC3) consortium. This paper describes and compares approaches to the initial development of seven I-Labs in order to gain an understanding of the development of research partnerships representing various implementation science designs.

**Methods:**

In April-June 2021, members of the ISC3 Implementation Laboratories workgroup interviewed research teams involved in I-Lab development in each center. This cross-sectional study used semi-structured interviews and case-study-based methods to collect and analyze data about I-Lab designs and activities. Interview notes were analyzed to identify a set of comparable domains across sites. These domains served as the framework for seven case descriptions summarizing design decisions and partnership elements across sites.

**Results:**

Domains identified from interviews as comparable across sites included engagement of community and clinical I-Lab members in research activities, data sources, engagement methods, dissemination strategies, and health equity. The I-Labs use a variety of research partnership designs to support engagement including participatory research, community-engaged research, and learning health systems of embedded research. Regarding data, I-Labs in which members use common electronic health records (EHRs) leverage these both as a data source and a digital implementation strategy. I-Labs without a shared EHR among partners also leverage other sources for research or surveillance, most commonly qualitative data, surveys, and public health data systems. All seven I-Labs use advisory boards or partnership meetings to engage with members; six use stakeholder interviews and regular communications. Most (70%) tools or methods used to engage I-Lab members such as advisory groups, coalitions, or regular communications, were pre-existing. Think tanks, which two I-Labs developed, represented novel engagement approaches. To disseminate research results, all centers developed web-based products, and most (*n* = 6) use publications, learning collaboratives, and community forums. Important variations emerged in approaches to health equity, ranging from partnering with members serving historically marginalized populations to the development of novel methods.

**Conclusions:**

The development of the ISC3 implementation laboratories, which represented a variety of research partnership designs, offers the opportunity to advance understanding of how researchers developed and built partnerships to effectively engage stakeholders throughout the cancer control research lifecycle. In future years, we will be able to share lessons learned for the development and sustainment of implementation laboratories.

**Supplementary Information:**

The online version contains supplementary material available at 10.1186/s12913-023-09128-w.

## Introduction

Implementation science relies on partnerships with stakeholders to study the implementation of evidence-based practice. These partnerships are based on a recognition that, to quote Dr. Lawrence Green, “if we are to have more evidence-based practice, we need more practice-based evidence” [1]. One novel approach to generating ‘practice-based evidence’ is the creation of “laboratories” comprised of practitioners and other stakeholders, partnered with researchers, to form research-ready environments in which to test implementation strategies. Implementation laboratories (I-Labs), such as those modelled in learning health systems or practice-based research networks [2], could enhance the field by expediting research on adoption of evidence-based interventions in diverse settings. Partnering members, such as community-based clinics and health-related organizations, may need support such as infrastructure or capacity building to enable them to engage in or serve as laboratories. Descriptions exist of research partnership models that leverage practice-based research networks or clinical data research networks, learning health systems, and community-engaged and community-based participatory research [3–15]. Prior studies describing the development of research partnerships using these different models have focused on one partnership or model at a time and lack a common framework or model that applies to different partnership designs. This prior work has highlighted several elements in those individual partnerships such as the consideration of data for different stakeholders, the importance of social issues of communication and decision-making, variability in methods of engagement from community-targeted to community-driven methods, navigating competing agendas, employing distributed leadership principles, the unique value of partnerships that extend outside of academic medical centers, methods for “cultural exchange” between academic and community groups, and shared goal setting [3, 5, 6, 8]. There have been few opportunities to compare the concurrent development of several such efforts to understand how partnerships generate and utilize data, develop social systems hand in hand with technical infrastructure, engage with community partners outside of academic medical settings, facilitate cultural exchange, and develop dissemination models for common goals of increasing the delivery of evidence-based cancer-relevant services. Comparing the development and structure of diverse implementation science laboratories may provide insight about key elements of research partnerships and the different forms these elements can take to align with a given research partnership’s mission and add value for its partners.

In 2018 the National Cancer Institute issued a funding opportunity announcement to support the development of Implementation Science research centers to build capacity for research on high-priority, emerging topics in cancer control. Core to these centers was the creation of implementation laboratories (Table 1).

**Table 1:** Funding Opportunity Announcement Excerpts

“*The purpose of this FOA is to promote the development of research centers that can build capacity to study high priority, emerging areas of cancer control implementation science,* build implementation laboratories, *improve the state of measurement and methods, and improve the adoption, implementation, and sustainment of evidence-based cancer control interventions.*”
“***Implementation Laboratory*:** *A collaborative research concept specific to the ISCCC. The "Implementation Laboratory" should reflect a collaboration between the Center awardee institution and an appropriate set of community and/or clinical sites. The collaborating sites may reflect diverse settings (e.g., oncology care, primary care, community services) but all are expected to share interest in and capacity to conduct research consistent with the implementation science theme of the Center. Each Implementation Laboratory should enable a range of studies focused on the adoption, implementation, sustainment, and de-implementation of various cancer control interventions. As appropriate, studies to be conducted may be observational, experimental, and/or quasi-experimental.*”
“*Implementation Laboratory* **to enable a range of observational, experimental and/or quasi-experimental pilot implementation studies to be tested within a set of clinical and/or community service settings that have shared interest in the Center theme, capacity for study participation, and engagement in improving cancer control across the continuum.**”

Between 2019 and 2020, seven implementation science centers were funded to develop research laboratories of community partners representing new or existing relationships and networks in the Implementation Science Centers in Cancer Control (ISC3) Consortium [16]. Along with the laboratories, centers were tasked with developing Implementation Studies Units to carry out innovative investigations in cancer-focused implementation science within the laboratory, and Methods Units to address gaps in related research methods. An ISC3 working group was formed with representation from each of seven centers to share learnings with other researchers about how the I-Labs were being developed. This paper describes how these implementation laboratories were initially operationalized across the seven centers from the perspectives of the research teams, including their goals, partnerships, data sources, engagement activities, methods for advancing health equity and how each center matched their design and activities to their unique models and purposes.

## Methods

### Study design

We collected baseline, cross-sectional information using methods based on a case study research approach [17]. The work was reviewed and approved as exempt human subjects research by Mass General Brigham Institutional Review Board and the Wake Forest Institutional Review Board.

### Interview protocol

From April through June 2021, two I-Lab workgroup members (GK and EH) conducted seven interviews, or one interview per Center. Interviews included 12 key informants total, between one and three individuals per center. Key informants were identified by workgroup members and included principal investigators, co-investigators, laboratory directors and project managers.

The interview guide was developed using guidance from prior literature examining real-world research partnerships and with the implementation science expertise within the workgroup (Supplemental File).The guide was shared with interviewees beforehand to allow for comprehensive scoping. The guide included closed- and open-ended measures to elicit a set of domains that were common and comparable across the varied I-Lab models. The guide referenced responses collected in an annual survey of each ISC3 center conducted by Westat. Closed ended questions assessed I-Labs’ purpose or focus, member engagement activities, laboratory data sources, engagement methods and dissemination tools. Open-ended questions further explored purposes, membership, data sources, member engagement, dissemination methods, approach to advancing health equity, and any changes to their design compared to what was initially proposed. Each interview was conducted by videoconference. Interviewee responses were recorded in structured field notes in REDCap [18]. The raw interview notes in REDCap were read independently by two researchers (GK and EH) who then met to discuss and reconcile any discrepancies in the accuracy of recorded responses.

### Analyses

Tables were used to compare closed-ended measures across centers. We performed inductive thematic analysis of open-ended item responses to identify additional domains that were common and comparable across I-Labs emphasizing design features and partnership-building activities that aligned with each I-Lab’s stated purposes. We used an iterative approach using dialogue to achieve consensus starting with consensus between the two coders. Each coder reviewed all reconciled notes and identified common domains, compared, and discussed the domains until a consensus was reached. Next, we reviewed the reconciled interview notes to generate descriptive case summaries of each ISC3 Center’s Lab using these domains as a framework [17]. Case summaries of each Center’s i-Lab were synthesized from July to September 2021. These summaries were shared with key informants. Key informants were invited to review and revise their center’s case description to ensure that it accurately reflected their design and methods. All centers provided revisions or additional content after review. Revised summaries were reviewed by the coding team to ensure clarity of each domain and to compare domains across sites.

## Results

The stated purposes of the seven implementation laboratories cover the intersection of cancer control with innovation, equity, community engagement, quality improvement, health policy and health equity (Table 2). Individual laboratories also specified other core purposes, i.e., building partnerships that span the cancer care continuum, behavioral economics, cost and value of cancer control interventions, early detection, health informatics, health information technology (IT), local adaptation, partnership building between communities and academia, power dynamics in partnerships, and health disparities. The domains that were common and comparable across sites, and which framed our case summaries included: member engagement in research activities, data sources, member engagement methods, dissemination strategies, and health equity.

**Table 2:** Implementation Laboratory Purposes, (*n* = 7)

	n (%)
Cancer Prevention	6 (85.7)
Equity	6 (85.7)
Innovation	5 (71.4)
Cancer Care	3 (42.9)
Survivorship	3 (42.9)
Community Engagement	3 (42.9)
Quality Improvement	3 (42.9)

Other specified laboratory purposes*:

Building partnerships that span the cancer care continuum, health policy, behavioral economics, cost and value of cancer control interventions, early detection, health informatics, health information technology (IT), local adaptation, partnership building between communities and academia, power dynamics in partnerships and health disparities

^*^Additional priorities mentioned by two or fewer centers

### Member participation in the research lifecycle

Across the phases of research, laboratories most commonly engaged members in identification of research priorities, study planning, and dissemination of results. Likely reflecting the focus of the funding opportunity on cancer control, primary care or FQHC organizations were the most common member types to participate in these activities. Community-based members such as public health departments and advocacy groups were the least common (Table 3). Within the research process – starting from the identification of priorities and extending to dissemination and capacity building – implementation laboratories least often engaged members in writing scholarly products such as manuscripts and grants.

**Table 3:** Engagement in research activities across the research lifecycle among I-Labs by member type (*n* = 7)

I-Lab member type, *n* = number of laboratories engaging with listed member type*	Identifying research priorities	Study Planning	Capacity building	Study Activities	Data analysis and interpretation	Writing scholarly products	Dissemination of results
Primary Care/Federally Qualified Community Health Centers, *n* = 6	6	6	5	5	5	4	6
Oncology/Cancer Center, *n* = 4	4	4	4	3	3	2	4
Hospitals/Health Systems, *n* = 4	4	4	4	3	3	1	4
Public Health/Health Departments, *n* = 4	4	4	3	2	2	1	3
Community Members/Community Organizations, *n* = 4	4	2	3	2	1	0	4
Other, *n* = 1	1	1	1	0	0	0	1

* The n for each member type is based on the implementation laboratory reporting that they did any of the listed research activities with that member type, it is possible that a laboratory partners with a member type but not on any of the listed activities or that a member type works with investigators on the listed activities but not through the implementation laboratory infrastructure

### I-Lab data types

Laboratories were involved in data collection for research purposes and for understanding or describing members and member needs. The most common data sources used by the laboratories are primary qualitative data, primary survey data and public health data systems (Table 4). Four of seven centers use electronic health records (EHR) data in their laboratories from primary care/FQHC, oncology/cancer centers, and hospitals/health systems. Two centers leverage a shared data reporting system/shared data platform with primary care/FQHC laboratory members. Six of the seven laboratories conduct primary qualitative data collection with their partners and five collect primary survey data with laboratory members. More data collection methods are employed with clinical partners in the implementation laboratories, including primary care/FQHCs, cancer centers and hospitals/health systems, compared to public health or community-based members.

**Table 4:** Data sources among I-Labs by member type^*^ (*n* = 7)

Member type, n = number of laboratories with this member type using each data source	EHR Data	Primary Qualitative Data	Primary Survey Data	Shared data platform/data reporting systems	Public Health Data Systems	Other primary research data	Other secondary research data
Primary Care/Federally Qualified Community Health Centers, *n* = 6	3	5	4	2	4	2	1
Oncology/Cancer Center, *n* = 4	2	3	2	0	2	2	1
Hospitals/Health Systems, *n* = 4	2	3	2	0	2	2	1
Public Health/Health Departments, *n* = 4	0	1	0	0	0	0	0
Community Members/Community Organizations, *n* = 4	0	2	1	0	1	0	0
Other, *n* = 1	0	0	0	0	0	0	0

^*^ The n for each member type is based on the implementation laboratory reporting that they work with the specified data with that member type, it is possible that a laboratory has a partnership with a member type that does not involve data sharing or collection or that a member type works with investigators with these data types but not through the implementation laboratory infrastructure

### Member engagement methods

The most-used member engagement methods are advisory boards, stakeholder interviews, and regularly scheduled communications with partners (Table 5). Across the seven laboratories, nine engagement tools were newly developed for ISC3, and 21 existing tools were leveraged for laboratory member engagement. There were a variety of descriptions of these tools in the interviews with no common vocabulary across centers.

**Table 5:** Engagement Methods used by I-Labs* (*n* = 7)

	Established method	New method
	n (%)	
Think tanks	0 (0.0)	2 (28.6)
Workgroups	2 (28.6)	1 (14.3)
Community engagement studios	3 (42.9)	0 (0.0)
Needs assessments	3 (42.9)	1 (14.3)
Community coalitions	1 (14.3)	0 (0.0)
Advisory boards	6 (85.7)	1 (14.3)
Stakeholder interviews	3 (42.9)	2 (28.6)
Regularly scheduled communications	3 (42.9)	2 (28.6)

^*^Interviewees were asked to classify each engagement methods as established or newly developed

### Dissemination strategies

All centers had planned activities for dissemination of I-Lab results. All centers used web-based products for dissemination and a subset used publications, learning collaboratives, talks and community forums, and social media (Table 6).

**Table 6:** I-Lab Dissemination Methods, (*n* = 7)

	n (%)
Web-based products	7 (100)
Publications	6 (85.7)
Learning collaboratives	6 (85.7)
Talks and community forums	6 (85.7)
Social media	4 (57.1)

### Health equity

Health equity is a common priority of all seven laboratories. All laboratories include settings serving and partnering with communities of color, diverse ethnicities, rural populations, uninsured individuals, individuals living in poverty, those facing adverse social determinants of health, disparate digital access, structural bias, or populations who are otherwise underrepresented in research and underserved by healthcare. However, the laboratories varied in their approaches to integrating or promoting health equity. Next, we present the I-Labs’ distinct approaches to: membership, member engagement, data sources, dissemination strategies and health equity.

## Individual I-lab descriptions

### The Harvard Implementation Science Center for Cancer Control Equity (ISCCCE)

#### Member participation in the research lifecycle

ISCCCE brings together researchers from the Harvard T.H. Chan School of Public Health and the Massachusetts General Hospital Kraft Center for Community Health with the Massachusetts League of Community Health Centers (Mass League) in a Community Engaged Research model. Mass League is the primary care association for federally qualified community health centers (FQHCs) across Massachusetts. The FQHC network was previously established primarily for clinical care. FQHCs have a history of participating in research, but the Harvard and MGH partnerships were newly developed to conduct implementation science research with the FQHCs.

#### I-Lab data types

Most Massachusetts FQHCs use a common data reporting and quality improvement digital platform that maps onto individual EHRs and this shared data platform enables participation in pilot project data collection with minimal data collection burden for partners. Laboratory membership evolves as FQHCs join or leave the shared data platform. Other sources of laboratory data are primary qualitative and quantitative data, EHR data and area-level public health data.

#### Member engagement methods

Member FQHCs have three different levels of engagement including: (i) those participating as pilot project sites, (ii) those actively participating in capacity building, our implementation learning community, and center-wide data collection to measure organizational context and cancer control practices in Massachusetts FQHCs, and (iii) FQHCs who are not actively involved in research with the center but who are invited to participate in ISCCCE implementation learning communities. Member FQHCs are provided with financial resources in accordance with the different engagement levels. Other engagement methods include a newsletter, regular meetings and a quarterly implementation learning community meeting. ISCCCE has a publication policy that requires implementation laboratory member representation on every academic publication. The center’s engagement strategies emphasize low-burden measurements and research methods. The implementation laboratory interfaces with the ISCCCE Methods Unit to design and evaluate these measures and methods. The Methods Unit is also leading efforts to understand how laboratory partners define and operationalize health equity, a primary focus of the center. The implementation laboratory further prioritizes principles of community-engaged research including fostering bidirectional benefits in partnerships and the co-design of projects and strategies to fit of the FQHC context.

#### Dissemination strategies

Dissemination methods include sharing results back to FQHC members via the quarterly learning collaboratives, talks and community forums, academic publications, and web-based products.

#### Health equity

The ISCCCE I-Lab employs five strategies to promote equity: 1) using low burden approaches to meet the needs of the federally qualified community health centers (FQHCs) context, 2) equity-focused strategies to produce efficiencies in implementation efforts such as pairing patient outreach activities rather than siloed outreach approaches, 3) developing data-informed methods to understand how define health equity and to adapt implementation strategies to improve equity, 4) exploring the role of outer context, the community-level measures of where patients and FQHC staff live and work and the impact of outer context on implementation, and 5) studying models of FQHC partnerships with community organizations.

### Building Research in Implementation and Dissemination to Close Gaps and Achieve Equity in Cancer Control (BRIDGE-C2) Center 

#### Member participation in the research lifecycle

The BRIDGE-C2 Center’s foci are equity, quality improvement (QI), primary care, community engagement, innovation, and health IT. The laboratory is built on a long-standing partnership with an existing practice-based research network (PBRN) made up of FQHCs and other safety-net primary care practices – all members of OCHIN, Inc., a non-profit IT collaborative serving safety net clinics [19, 20]. PBRN members include > 650 clinic sites run by > 120 organizations; all share a single instance of the Epic© EHR, and remotely provided IT support for that EHR. Research is usually conducted at the clinic site or organization level.

#### I-Lab data types

The implementation laboratory data infrastructure leverages OCHIN’s clinical data warehouse, which includes clinical data from the EHR with community-level social determinants of health data (i.e., EHR data are geocoded and linked to data on community-level factors with potential to impact health) [21]. Individual-level linkages can also be made with other data sources, such as insurance claims and cancer registry data. Primary qualitative data is also collected from laboratory members on a project-specific basis. The BRIDGE-C2 Laboratory created an interactive, longitudinal practice surveillance tool using EHR data visualized in Tableau software. This tool enables the overlay of longitudinal trends in cancer control with concurrent events (e.g., new IT tools, QI initiatives, FQHC reporting requirements, payment incentives) to study the temporal relationships between these events and changing trends in cancer care quality. Scientists in the BRIDGE-C2 Methods Unit partner with the Laboratory to review this surveillance data regularly. This facilitates pilot selection and fosters discussion on learnings from completed pilots.

#### Member engagement methods

A broad range of engagement activities occur with various stakeholder groups, leveraging a culture of engagement across the organization. Specific activities include monthly webinars, a monthly newsletter, a grand rounds series, research, and QI standing meetings, and project-specific meetings. Engagement is bi-directional and includes patients, clinicians, and health system leaders as co-investigators on research studies as well as the integration of researchers into clinical, IT, and leadership workgroups. An annual learning forum provides a platform for sharing best practices and new knowledge. OCHIN established a patient engagement panel (PEP) in 2012 as an ongoing, unique research engagement group [22]. Individual projects also recruit patients, clinicians and other stakeholders to serve as advisors, and OCHIN’s ‘provider builder’ program enhances these partnerships by educating safety net providers about optimizing EHR use and tool development.

#### Dissemination strategies

Where relevant, materials are created highlighting practical implementation science methods developed by Laboratory pilots [23]. The surveillance data dictionary, co-developed between the Laboratory and the Methods Unit, is a durable, updatable product designed to ensure consistency of definitions across pilot projects.

#### Health equity

BRIDGE-C2 works with a large national network of FQHCs serving under-insured and traditionally under-represented patients. Their goal is to support quality improvement work in FQHCs and other safety net settings, which traditionally have many fewer resources and more vulnerable patients than clinics serving commercially insured patients and large integrated health systems. The BRIDGE-C2 laboratory interfaces with the largest existing EHR-embedded database of patient-reported social risk information, with > 1,000,000 documented patient-reported social risk screening results from > 800,000 patients. BRIDGE-C2 can also study the impact of community-level determinants based on OCHIN’s ongoing work establishing individual patient geocoding and linkage to community-level data.

### Colorado Implementation Science Center in Cancer Control (Colorado ISC^3^)

#### Member participation in the research lifecycle

The Colorado ISC3 Implementation Laboratory aims to take cancer prevention strategies that work in urban areas and implement them in rural settings. The laboratory is built with four existing primary care PBRNs which include urban and primarily rural PBRNs, a Cancer Center and the American Academy of Family Practice National Research Network. There are different levels of membership based on engagement. All PBRNs are members while a subset of practices within these networks are currently participating in pilot projects.

#### I-Lab data types

Laboratory data comes from shared data platforms, public health data systems, and primary qualitative and survey data.

#### Member engagement methods

Members engage through community engagement studios, needs assessments, advisory boards including a recently formed rural cancer advisory board, regularly scheduled communications, and stakeholder interviews. Interviews with members align with the core purposes of the laboratory: cancer prevention, cost, and value-based care. Interviews aim to understand how practices weigh cost and value to inform the practice’s decisions about what cancer prevention activities they focus on and participate in. The Implementation Laboratory leverages the PBRNs’ resources such as existing advisory boards and network practice facilitators to co-create implementation strategies. In this way, the individuals conducting practice facilitation for pilot projects, currently focusing on shared-decision making for cancer control, have a longstanding relationship and on-the-ground experience with the pilot practices.

#### Dissemination strategies

Dissemination strategies are diverse and include learning collaboratives, talks and community forums, academic publications, web-based tools to facilitate the use of Dissemination and Implementation theories, models, and frameworks, social media, posters and other printed information, creation of e-learning modules, and a PBRN convocation which is a convening of practices, community-members, researchers, and other partners to share successes and struggles of the network.

#### Health equity

The Colorado ISC^3^ focuses on the impacts of social determinants of health and targets implementation with rural and frontier communities in Colorado that face disparities in cancer outcomes. In addition, Colorado ISC^3^ actively engages rural stakeholders to understand their needs and priorities and ensure principles of equitable community engaged research are followed in center projects. Two prominent examples are the recent formation of a rural cancer advisory board, and a needs and priorities survey conducted throughout the Colorado ISC^3^ catchment area focused on determining community perceptions of gaps in cancer prevention and control activities and directions center activities should take.

### The Optimizing Implementation in Cancer Control Center (OPTICC)

#### Member participation in the research lifecycle

The OPTICC Implementation Laboratory includes networks and systems that span the cancer control continuum, including a practice-based research network, a large integrated health system, a learning collaborative of federally qualified health centers, a network of hospitals, a network of cancer treatment centers, a rural-serving cancer treatment center, and two public health departments [24]. OPTICC, a partnership between the University of Washington, Kaiser Permanente Washington Health Research Institute, and the Fred Hutchinson Cancer Research Center, brings those partners together for the first time, building on the investigators’ history of collaboration with most of the partners. Implementation Laboratory members are organized with a point person representing each of these individual partnerships. The point person is someone with a central view of the organization/health system or network.

#### I-Lab data types

Implementation Laboratory data derives from primary qualitative and survey data as well as other secondary research data.

#### Member engagement methods

The research team systematically communicates with the point person to identify research priorities in their organization/network, share OPTICC communications, and identify partners who could be a good match for specific research projects. The membership is open to new members, but within the first two years of the I-Lab the membership has been stable. Member engagement relies on a biannual newsletter about OPTICC progress, annual interviews with each point person about their organization or network’s priorities, implementation barriers and pandemic-related challenges. At any given time, a subset of members is actively engaged in pilot projects, and all members are involved in engagement activities. OPTICC also organizes an annual meeting bringing together Implementation Laboratory members and researchers and the OPTICC team strives to participate in members’ annual meetings or conferences.

#### Dissemination strategies

The Implementation Laboratory disseminates research and results from the center through established learning collaboratives in their member networks, a center website, and a series of 1–2-page research briefs to engage a broader audience than is achieved with standard research publications.

#### Health equity

The Optimizing Implementation in Cancer Control Center (OPTICC) center chose laboratory partners that serve populations experiencing health disparities due to one or more social determinants of health. The laboratory seeks to understand how their partners understand and address equity in their work through their annual qualitative interviews as well as a project examining how social needs data are collected and used. Pilot proposals in OPTICC are rated on their potential to impact health equity. The center works with laboratory members to understand capacity issues and the need for adaptations to make interventions feasible and/or to better fit the needs of patients served.

### Implementation and Informatics – Developing Adaptable Processes and Technologies for Cancer Control (iDAPT)

#### Member participation in the research lifecycle

The iDAPT Implementation Laboratory was built with existing and expanding partnerships at Wake Forest and the University of Massachusetts. These partnerships leveraged the NCI funded National Community Oncology Research Program (NCORP), existing hospital systems, and new partnerships within these systems. These partnerships were primarily developed for clinical care, QI, and training with some work focused on research. The iDAPT center enabled the expansion of implementation science research within these partnerships.

#### I-Lab data types

Implementation Laboratory data comes from EHRs, primary qualitative and survey data collection, hospital QI data, pilot project data and public health data systems.

#### Member engagement methods

Members are engaged at varying levels based on participation in pilot activities or contributing data and participating at the health system or individual practice level. For example, some individual practice participation may be at the health system level as part of high-reach, low-touch implementation strategies. Membership is evolving as members join or leave the networks and level of participation is fluid. Level of participation ranges from helping to prioritize future projects, sharing information about innovations at the practice level with the potential for scale-up, and participation in capacity building such as trainee engagement or member site staff learning about implementation science and learning health systems. iDAPT uses diverse engagement methods for working with its members including engaging with health system and practice leadership, working to align research interests with operational interests, and providing resources to member clinics who have actively engaged to continue their participation. This engagement work is achieved through workgroups, needs assessments, advisory boards, stakeholder interviews and regularly scheduled communications.

#### Dissemination strategies

Results are disseminated through learning collaboratives, posters, and other presentations in local institutions, academic publications, and web-based products.

#### Health equity

The iDAPT Implementation Laboratory also focuses on the impacts of social determinants of health with particular attention to the digital divide. In their laboratory, they see an approximately 60% difference in patient portal access between patients of high and low socioeconomic status. iDAPT is also exploring the roles of healthcare system bias and trust of the healthcare system in the digital divide, beyond just internet access.

### Washington University Implementation Science Center for Cancer Control (WU-ISCCC)

#### Member participation in the research lifecycle

The WU-ISCCC is built on new and existing partnerships including partnerships for reducing cancer disparities and promoting health equity, and a network of community-based research partners in the Siteman Cancer Center catchment area in Missouri and Illinois. Much of the laboratory’s work arose from a long-time community-based cancer disparities program for research, outreach, and service. In addition to this program, the Implementation Laboratory and its members have engaged new partners from across our catchment area, and new academic colleagues. WU-ISCCC continues to work with partners to identify gaps where new partners were needed such as organizations that address social determinants of health including housing organizations and community development advisors within the federal reserve bank. These new partners, in turn, help to broker new relationships and engagement. Member types include primary care, community advocates, community organizations, community health centers, hospitals and healthcare systems and academic partners.

#### I-Lab data types

Laboratory data sources include public health data, EHR data, and primary qualitative and survey data from pilot projects.

#### Member engagement methods

Membership is open to new members and engagement is achieved through interactive think tanks, among other engagement activities. These events bring members together to build and strengthen relationships and are built on a premise that all members bring expertise and critical insights. Think tank activities include brainstorming pilot projects, reviewing pilot proposals, sharing back research findings, getting to know community priorities, and linking researchers to community-based partners (and vice versa). One of the first products of the think tanks was a set of principles, informed from partners and the Design Justice approach, that provides a framework for giving as many people as possible a chance to participate and have their voices heard through different modalities and schedules [25]. WU-ISCCC also produces data briefs with a snapshot view of partner communities self-identified needs in areas related to social determinants such as transportation, housing, and food insecurity based on public health surveillance data. In this way the researchers in the center provide community partners with relevant data and resources in a way that is helpful for them. Think tanks are complemented with needs assessments and advisory boards.

#### Dissemination strategies

Dissemination of results used multiple modes, to echo the multiple modes for engaging in think tanks. Dissemination is achieved through learning collaboratives, talks and community forums, academic publications, web-based products, and social media.

#### Health equity

The WU-ISCCC Implementation Laboratory designed their member engagement activities with equity in mind to allow members to participate to the extent that they are able and interested. They do this by accommodating in-person, video, full-time, and part-time participation in their think tanks, and by using multiple channels for communication and conversation. This design decision considered power dynamics and aimed to offer more ways for people to contribute instead of giving more voice to the people who have more time to share. The WU-ISCCC team also examined think tank data from academic partners and community partners by comparing and contrasting data from these two groups and pushing community voices to the top of the priority list for pilot projects. They monitor the number of academic partners versus community partners in the think tanks and observe think tank activities to learn how to best prepare all participants so that multiple voices can be heard.

### The Penn Implementation Science Center in Cancer Control (Penn ISC^3^)

#### Member participation in the research lifecycle

The Penn ISC^3^ Implementation Laboratory is built on the existing cancer care network at Penn Medicine including six hospitals and 12 outpatient cancer clinical sites that comprise the Penn Medicine Cancer Service Line and which are part of the Penn’s Abramson Cancer Center, a National Cancer Institute designated Comprehensive Cancer Center. As part of the academic health system, research programing has previously been embedded within the Penn Medicine Cancer Service Line. The Penn ISC^3^ newly brings this network for cancer care delivery into the nexus of behavioral economics and implementation science research.

#### I-Lab data types

The Penn Medicine Cancer Service Line uses a common EHR, Epic (one hospital of the six is on a different instance of EPIC than the others). The common EHR facilitates implementation science research.

#### Member engagement methods

Research within the Penn ISC3 is largely conducted through the EHR and embedded in the real-world operations of the health system. A particular emphasis of the Penn ISC3 is to test implementation strategies informed by behavioral economics on clinicians, patients, or both, compared to usual care. Penn ISC3 studies are implemented across the implementation laboratory and all member sites are eligible. Members are engaged throughout the research life cycle, with member priorities informing pilot project selection.

#### Dissemination strategies

The Penn ISC3 laboratory disseminates research findings to academic audiences and lay people through talks and community forums, publications, web-based products, social media, and op-eds. Although the center is not policy focused, dissemination efforts include the policy community with the goal of impacting policy change.

#### Health equity

The Penn ISC^3^ promotes equity by engaging with Penn’s hospitals and outpatient clinics that serve racially, ethnically, and socioeconomically diverse patient populations in urban and rural settings. Their center considers the equitable reach, effectiveness, and implementation of all activities and the heterogeneity in historically underrepresented groups across rural and urban settings. Their Implementation Laboratory examines who is included and who is not represented in studies or efforts and examines patient outcomes by race, ethnicity, income, and rural/urban residence.

### Laboratory changes from initial proposals and other challenges

The COVID-19 pandemic created challenges for all ISC3 laboratories. For most, member engagement methods had to be adapted to align with social distancing policies, moving meetings from in-person to virtual formats, which impacted relationship-building with new partners. For laboratories with public health-focused members such as public health departments or community health centers, the pandemic affected representation of member organizations. Laboratories described being careful not to strain partners at the frontlines of pandemic response. For some laboratories, the pandemic impacted laboratory members’ participation in the pilot projects. Impacts included changing the priority of pilot projects to match the shifting needs of healthcare providers, modifying pilot project interventions to include interventions that could be delivered by telehealth, and altered timelines.

In addition to pandemic-related challenges, laboratories described changes in their laboratory designs or activities. These included working to develop new partnerships by engaging with practice or health system leadership, working to align with health system operational interests, modifying engagement tools by working with researchers to produce data in a format that is helpful and relevant for community partners, identifying where new partnerships or growth was needed to meet center goals, and changing center priorities to reflect consortium-wide priorities of equity and engagement with junior researchers.

## Discussion

The ISC3 I-Labs include diverse partnerships, models, and settings with the shared goal of supporting implementation research across the cancer control continuum through robust member engagement with a focus on health equity. All laboratories are designed to advance implementation science methods and improve cancer prevention and treatment in diverse healthcare and community settings via research. The ISC3 consortium presents an opportunity to compare several such partnerships. This work adds to our understanding of the development of implementation science models of research partnerships beyond the single-site or single-network studies previously described in the literature. This paper sought to describe and compare key domains of the initial development of the seven I-Labs across seven sites as a baseline assessment from the perspective of the research teams. Going forward, this group will be equipped to examine the continued development and sustainment of the I-Labs of varied implementation science partnership designs from the broader perspectives of academic researchers and their community and clinical partners to identify best practices in areas ranging from partnership strengthening and maintenance, capacity building, and stakeholder engagement in the research lifecycle.

We identified several common priorities among the seven ISC3 I-Labs. All were built around bidirectional partnerships between researchers and diverse laboratory settings with shared processes to identify priorities and methods. Initial steps for all I-Labs would be to have a shared understanding of how the laboratory is structured and how all partners will interact as well as early collaborative work that defines clear goals for what all partners want to achieve and what roles are involved. A goal of building bidirectional partnerships with implementation laboratory members is to engage with partners throughout the research lifecycle.

We identified heterogeneity in member engagement methods and stakeholder involvement in different phases of the research lifecycle. There were a variety of labels that the centers used to describe their member engagement methods and no common vocabulary has emerged for example for advisory groups, engagement studios, or think tanks. These labels may represent the varied partnership models (e.g., PBRNs, community-engaged research, or learning health systems) or other nuances in how partnerships are conceived and implemented. Adopting a common vocabulary may enable consistent and transparent methodology, the evaluation of when certain methods work best, and their impact across centers. Within the research lifecycle, among our laboratories, partners were least often engaged in the production of scholarly products. This may reflect partners’ priorities or an area where laboratories could do more to apply established methods for community-partnered research [9, 26, 27].

There was also variability in the types of data used in I-Labs depending on the type of partnership. Having shared clinical data systems enabled learning health system models and the study of digital implementation strategies in practice-based research network models. There were more data types collected with clinical partners compared with community partners which primarily included qualitative data, surveys, and public health data. Innovative ways to expand the use of varied data sources with community partners, such as leveraging technology-enabled data sources with community partners including case management or other client data software, when it exists, may strengthen our ability to study the impact of community-stakeholders on cancer control intervention implementation and outcomes.

A limitation of this work is that it was set in the context of the pandemic. All laboratories described how the pandemic highlighted the need for flexibility in their approach to accommodate the competing priorities of their partners at this time. The pandemic required laboratories to accommodate changes to pilot studies, virtual member engagement and the competing priorities of members working at the frontlines. Moving forward, the laboratories are positioned to be able to study whether any pandemic-related changes to our approach to community-engaged research will be sustained. The comparative study of I-Lab community or clinical partner perspectives on initial I-Lab development is an important next step, as is sustainability of these partnerships. However, these topics are beyond the scope of this study. Data collection was not supported by dedicated funds or staff, such as funding for transcription, so that the team relied on field notes without verbatim interview transcripts. However, we felt this simple, inexpensive method which allows interviewers to collect their interpretations and connections they make from the interview data was effective in this project [28].

Among these seven cancer control implementation laboratories, various approaches are aimed at identifying and addressing patient-level and system-level barriers to cancer prevention and treatment. The engaged laboratories within the ISC3 consortium and the methods developed aim to help researchers in furthering our understanding of different implementation barriers in a systematic way, and thus to improve the adoption, adaptation, and sustainability of evidence-based interventions throughout the cancer care continuum – as well as the de-implementation of ineffective interventions. While methods are evolving with ISC3 I-Labs, they may also serve as blueprints for the development of future implementation laboratories. The ISC3 presents replicable models for building implementation science capacity in diverse laboratories; the results presented here point to some of the infrastructure, training, and knowledge needed in robust clinical and community laboratory partnerships to accelerate stakeholder-engaged research. It also speaks to the need to ensure that implementation science terminologies and conceptual models are made as applicable and relevant as possible to a wide variety of laboratory settings, partnership models, and community stakeholders.

## Supplementary Information

**Additional file 1:** **Supplemental File.** I-Lab interview guide

## Data Availability

The datasets used and/or analyzed during the current study are available from the corresponding author on reasonable request.
