# Supplementary material for: Creating research-ready partnerships: the initial development of seven implementation laboratories to advance cancer control
Source: BMC Health Serv Res. 2023 Feb 21;23:174. doi: 10.1186/s12913-023-09128-w (PMC9942028; doi:10.1186/s12913-023-09128-w)
Supplement: Supplementary file 1 — Additional file 1: Supplemental File. I-Lab interview guide [file 12913_2023_9128_MOESM1_ESM.pdf]

Supplemental File. I-Lab interview guide

We would like to learn from each i-lab about their focus, purpose, and formation to identify common “themes” and unique aspects of the different labs including the membership structure and different levels at which we ask/expect stakeholders to engage and how this aligns with the lab’s purpose. We plan to use this data to produce a manuscript comparing I-Lab purposes and formation processes. Your responses will be collected as field notes in a REDCap form.

Please indicate your lab’s purpose or focus. [check all that apply, indicate which are primary focus]

□ Cancer prevention	□ Equity
□ Cancer care	□ Quality improvement
□ Survivorship	□ Innovation
□ Health policy	□ Other____________
□ Community engagement	

Is your I-lab built on an existing relationship (e.g., existing cancer network, hospital system or PBRN) or new relationships?

New: please describe_________________

Existing: please describe______________

Tell us about how or what parts are new vs. existing?

Did the existing infrastructure have a research purpose or another purpose? Please describe

How do you define membership?

Is your I-Lab membership static or does the membership evolve over time (e.g., members coming and going)?

Briefly describe the activities of members in your lab:

In the prior Westat survey you listed [X] members or type of members [list members]

For each member or type of members, note whether they are new for this grant vs. established working relationships

Member	Description of partnership with member: e.g., is it new for the grant or longstanding established research relationship

Do you have different categories of membership in your I-lab model? Y/N

If Y, how are the categories organized?
Probe:

- By type of organization (e.g., oncology, primary care, public health, CBO)
- By type of engagement (e.g., pilot study site, capacity building, CAB)
- Other_________

What aspects of your research do you plan to engage members in/have you engaged members in?

	Primary care/FQHCs	Oncology, cancer centers	Hospitals and health systems	Public health, health departments	Community members & organizations	Other:_____
Identifying research priorities						
Study planning						
Study activities (e.g., data collection, study visits)						
Data analysis and interpretation						
Writing manuscripts/grants/other scholarly products						
Dissemination of results						
Capacity building						

Has your I-lab design changed at all from what you initially proposed/planned? Y/N
If yes, how, and why has it changed.

In what ways is your lab structured to address equity?

In what ways do your lab activities address equity?

How has your I-lab operationalized equity in your work?

Have your I-lab activities changed at all from what you initially proposed/planned? Y/N
If yes, how, and why have they changed.

What methods/tools do you use to foster strong relationships with your lab members?

Where does your lab data come from?

	Primary care/FQHCs	Oncology, cancer centers	Hospitals and health systems	Public health, health departments	Community members & organizations	Other:_____
EHR data						
Primary qualitative data						
Primary survey data						
Shared data platform/ data reporting systems						
Case management or other client management software						
Public health data systems						
Public use data						
Other primary research data______						
Other secondary data______						

What methods/formats do you use for engaging stakeholders in identifying research priorities?

	Established method	Newly created method
Think tanks	◻	◻
Workgroups	◻	◻
Community engagement studios	◻	◻
Needs assessments	◻	◻
Community coalitions	◻	◻
Advisory boards	◻	◻
Stakeholder interviews or focus groups		
Regular communications (scheduled calls or meetings)		

What methods/formats do you use or plan to use for engaging stakeholders in dissemination of results?

- ☐ Learning collaboratives
- ☐ Talks and community forums
- ☐ Publications
- ☐ Web-based formats/products
- ☐ Social media
- ☐ Other______________

Tell us a little bit about why you chose this/these method(s) for dissemination?
